# Policies to Promote Medical Tourism in Korea: A Narrative Review

**Published:** 2018-08

**Authors:** Byung Ro SEO, Sam-Hun PARK

**Affiliations:** 1. Dept. of Global MICE, Konkuk University, Seoul, Korea; 2. Asia Contents Institute, Konkuk University, Seoul, Korea

**Keywords:** International meditour coordinator, Medical sector, Medical tourism industry, Korea

## Abstract

**Background:**

There is a growing global demand for medical tourism while more people are moving across borders in Asia, offering new opportunities to the high-value medical tourism market. The purpose of this study was to provide grounds for promoting the International Meditour Coordinator (IMC), issued as a national license in Korea and to provide the evidence for the efficient use of the IMC in the field of health care.

**Methods:**

We examined the trend of professional manpower in the rapidly-changing Korean medical market by analyzing the operational status of professional manpower in order to promote medical tourism, which began in 2009 in Korea. We also analyzed the problems of the national qualification system for the IMC and sought to improve the professionalism and usability of the national qualification system by improving the quality satisfaction of the professionals who support the growth of the medical tourism industry.

**Results:**

In Korea, IMCs are responsible for detailed support services in clinics, support to tourists, medical tourism marketing to support the advancement of domestic and international medical institutions in each market, medical tourism counseling, risk management, and administrative work, thereby contributing to the development and enhancement of competitiveness in Korea’s global healthcare industry.

**Conclusion:**

To support the growth of the medical tourism industry, which is a global trend, and increase user satisfaction with the quality of medical tourist services, it is urgently necessary to establish human resource management policy guidelines in the medical tourism industry based on the current operational status of professional human resources and the future prospect of supply and demand in the sector.

## Introduction

The global economy has been rapidly changing through inter-industrial convergence centered on the fourth industrial revolution. The future will be an era of strategic thinking where the invisible brain game and strategies determine the directions of a country (1). Futurologists forecast that the medical tourism industry will be a promising sector and believe that it will be the highest value-added service industry through inter-industrial convergence in the era of the Fourth Industrial Revolution (2). In this regard, many governments across the world have been eager to nurture and develop the medical tourism industry. The Korean government has also designated the tourism industry as a national strategic industry and, accordingly, the central and local governments are making various efforts to nurture it and develop more diversified tourism destinations and packages.

The Korea Health Industry Development Institute (KHIDI), a government-affiliated institution under the Ministry of Health and Welfare, defines health tourism as a “business that aims to contribute to the national economy by identifying and developing tourism resources available in the health sector and developing the health tourism programs (packages) combined with these tourism resources, aiming to develop related industries and attract foreign tourists to earn foreign currency” (3).

The size of the global medical tourism market increased about 2.5 times from 2004 to 2012 by approximately USD 10 billion, and it is expected to reach approximately USD 33 billion by 2019 (4). In particular, some of the major Asian countries, including Thailand, Singapore, India, Malaysia, and the Philippines, have grown as major destinations and global medical tourism hubs presenting benefits, such as low medical costs, high quality medical services, short waiting times, and tourism packages combining relaxation and tourism (5). In Thailand, one of the leading medical tourism destinations, the number of medical tourists increased from USD 630,000 in 2003 to 2.5 million in 2013, and the country earned annual profits of USD 4.3 billion (equivalent to KRW 4.7 trillion) in 2013 in the medical tourism sector. The Asian medical tourism market size increased from 4.3 million users in 2012 to 6.8 million users in 2015, and the total revenue of the medical tourism industry grew dramatically from USD 7.3 billion in 2012 to USD 14.7 billion in 2015 (6).

Factors for the growth of the global medical tourism market can be attributed to the dissatisfaction of the consumers from developed nations about the medical standards, long waiting times, aging population and medical technology development.

As the burden of medical expenses increases, the government and consumers are exploring alternatives to medical expenses, and medical tourism is attracting attention as a major strategic industry in the world (7). The reason medical tourism has grown as an industry is that many countries have supported it with policy efforts at the governmental level to generate high added value (8).

According to the Korea Health Industry Development Institute (KHIDI), it has been reported that the number of foreign patients visiting Korea has been increasing every year, from around 27,400 persons in 2008 to 159,464 in 2012 and 364,189 in 2016 (9). Thus, with the increasing number of foreign patients visiting Korea and the development of the Korean medical tourism industry, the role of IMCs in attracting and providing services to international patients is becoming increasingly important.

In 2013, Korea became the first country to introduce the national licensing system of International Meditour Coordinators. IMCs, also known as medical tourism marketers, medical tourist service coordinators, or medical tourism interpretation coordinators, are professionals who help foreign patients with their schedules. They provide a range of services right from helping patients travel from their home country to Korea, facilitating their treatment including surgery, to easing their discharge from hospital and travel back to their own country (10).

We examined the status of Korea’s IMC qualification system and the problems of the national qualification examination system, and suggests a direction for governments around the world for establishing policies to enhance competitiveness in the medical service sector.

## Results and Discussion

### Overview of the International Meditour Coordinator Licensing System of Korea

As international competition for medical services has been intensifying in many countries around the world, there has been an effort to strengthen the competitiveness of attracting medical tourists. In January 2009, the “Registration Act for Foreign Patient Registration (Article 27-2 of the Medical Law)” was passed to attract and obtain medical tourists (11).

The medical tourism market in Korea began to take off after the revisions to the Korean medical laws of May 2009 and January 2010 provided the legal grounds for the promotional activities of hospitals designed to attract foreign patients and the operation of accommodation by medical corporations as an incidental business.

As the medical tourism industry has been revitalized and the demand for the professional work-force has increased, research on tourism education courses have been conducted since the 1980s and research for the training of the professional manpower in the tourism industry have been actively conducted since the 1990s (12). The study emphasizing the importance of on-the-job training in tourism-related education has begun to raise the importance of training courses that include industrial adaptation of tourism education (13). This has raised awareness of the importance of the field and the importance of consumer - centered education in the curriculum as well as the range of the study in the tourism industry.

Therefore, in 2013, the Korean government was the first in the world to introduce an international system of medical tour coordinators, so that international patients could comfortably visit Korea for tourism and medical services, and began to aggressively stimulate professional instructors of educational programs to obtain the medical tour coordinator license. In the medical tourism industry where its target is the global market, language proficiency in the mother tongue of the overseas patients are essential and of utmost importance. The IMC is the core of the medical tourist attraction business based on medical knowledge and ability to translate so that medical tourists can receive high quality medical services, and medical service providers can effectively manage customers (14).

According to the type of duty, medical tourism professionals can be broadly classified as coordinators, marketers, international specialized medical doctors, and nurses. IMCs perform medical tourism marketing to support the cross-border advance of domestic and international medical institutions, medical tourism counseling, risk management, and administrative work, as well as provide detailed clinic services for patients and tourism support. They can be also classified as international patient care coordinators who assist patients involved in surgeries within the medical institution, and medical tourism interpretation coordinators who assist in accurate communication between the patient and the medical staff (15). The duty types of such medical tourism professionals are described in Table 1.

**Table 1:** Duty types of medical tourism professionals

***Classification***	***Job Type***	***Duty Type***
Coordinator	Medical tour coordinator	Plan and operate the overall process of medical tourism and provide related services to medical institutions and customers
	International clinic service coordinator	Establish the overall plan and operate regarding the provision of clinical services to international patients based on medical expertise
	International patients care coordinator	Perform specialized patient care within the medical institution for intensive-care patients who have gone through surgery
	Medical tourism interpretation coordinator	Provide interpretation service based on medical expertise and terminologies so that the patient and the medical staff can accurately communicate with each other
Marketer	Medical tourism promoter/marketer	Establish and implement promotional strategies and plans for medical tourism packages
	Medical tour package planner	Expert for planning and development of medical tour packages
	Medical tourism consultant	Provide consulting services including establishing infrastructure for the medical tourism industry and implementation strategies by helping medical institutions to enter into foreign markets and developing and operating systems to attract international patients

The Korean government has introduced the international system of medical tour coordinators under the national technical qualification system to nurture medical tourism professionals. The national technical qualification system evaluates job performances according to certain criteria for those working in the industry and those who want to work in the industry. When they reach a certain level, they are granted certification and recognition. This aims to improve the treatment and social status in terms of pay and promotion in the industrial society, and to contribute to productivity improvement with the ability of certain skills and functions (16).

Moreover, IMCs receive their license based on the technical qualification examination results so that they can easily adapt themselves to the industrial society. Moreover, the functions and roles of Korea’s competitive and differentiated medical services can appeal to international patients, and the development plan to boost the medical tourism industry can be established (Table 2).

**Table 2:** IMC Test Subjects and Test Method

***Classification***	***Written test***	***Practical test***
Test type	Multiple choice test	Short-answer/essay test
Test method	Select one from 4 choices	Write down answer to each question (for appx. 2 hours and 30 minutes)
Total no. of questions	Total 100 questions	20 short-answer/essay questions
Test subject	Health and medical tourism administration, health and medical service support management, health and medical tourism marketing, tourist service support management, understanding of medical terminology and diseases	Health and medical tourism practice (medical tour planning, medical tour implementation, customer satisfaction service)
Evaluation method		Central grading method
Total score and passing grade	40 points or higher out of 100-point-scale,	60 points or higher out of 100-point-scale
	Average score of 60 or higher in all test subjects	
Test provider	Human Resources Development Service of Korea	
Remarks	Strengthening on-the-job training after passing the national qualification, 20 hours a year (Educational institutions and government agencies accredited by national authorities)	

Source: Adapted from Human Resources Development Service of Korea (2018), *Guide to National Technical Qualification Examination*, Human Resources Development Service of Korea, pp.: 1–8. [Korean Book]

The number of people who passed the national qualification examination for the IMC license was 49 in 2013, 44 in 2014, 154 in 2015, 222 in 2016, and 140 in 2017. In the meantime, the number of international patients visiting Korea showed a rapid increase from 60,201 in 2009 to 159,464 in 2012 and 364,189 in 2016. Considering this trend, it is expected that the number of IMCs to be employed in the tourism service sector will consistently increase in the next decade.

Moreover, it is not a private qualification license but a national technical qualification license, so the license holder can get an advantage in the hiring process of many government agencies and public organizations in the medical and tourism sectors, as well as in hospitals and travel agencies. The IMC refers to a person who speaks one or more foreign languages and who completed required training courses. They are trained to obtain a substantially high level of medical expertise through specialized courses and to help the medical staff and the medical tourist to understand and empathize with each other with a great proficiency of foreign languages.

### Main Activities of the International Meditour Coordinator (IMC)

The IMC provides specific medical services for attracting and managing foreign patients in the internationalized medical market, tourism support, medical tourism marketing, medical tourism consultation, risk management and administrative work that can support the advancement of domestic and overseas medical institutions. Through this work, it is possible to improve the development and external competitiveness of the global healthcare industry in Korea (10).

The main activities of the IMC are as follows: 1. inquiry and counseling of medical tourists and collecting information on diseases. 2. Consultation of itineraries of medical tourists by planning their stay. 3. Medical tourism reservation and visa work. 4. Reservation of accommodation, flight, additional service and patients. 5. Hospital visit, inspection and treatment. 6. Payment and certificate issuance, sightseeing and shopping guide 7. Stay extension procedure. 8. Departure, after-care and secondary planning (17).

Medical tourism service is a profession that requires high level of knowledge and skill and has to meet qualification requirements by a certain regulation. The demands of cultivating talented people with creative skills and scientific theories are linked to qualifications, and international IMC service education and qualification standards are very important. However, the labor supply model in Korea is a bottleneck due to the concentration of young manpower in the form of college graduates at the entrance of the labor market. As a result, there is a trend of working at jobs which require less qualifications due to educational inflation, and there is an increase in employment unrelated to academic majors.

Therefore, in order to revitalize the supply of professional human resources for the IMC, professional education must be supported in order to boost pride of professionalism in career selection similar to countries such as Germany and the USA.

### Career Path for International Meditour Coordinators

As the market is expanding, IMCs have been entering into a wider range of areas. The medical tourism sector can be broadly classified into the health and medical care sector, the tourist service sector, and freelance coordinators, and in the health and medical care sector, they can work at the international clinic center at medical institutions, individual clinics, or associations of hospitals.

In the tourist service sector, they can get a job at domestic and international travel agencies, tourism associations, public organizations in the tourism sector, and hotels as tour package planners, tour guides, and instructors, among others.

To look at each of the medical tourism organizations, IMC license holders can get a job at the international cooperation department or at the international clinic center of hospitals including university hospitals, general hospitals and medical centers, as well as at medical consulting agencies, public health sector, travel agencies, hotels, agencies, etc. (10).

**Fig. 1:**
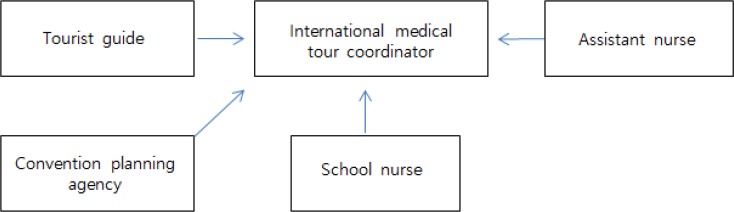
Relations of IMC Licensing

As indicated in Fig. 1, currently in the tourism-related sectors, the work of tourist guides is similar to that of IMCs, and IMCs are qualified to apply for the examination to obtain the tourist guide license. Therefore, it is helpful to expand the scope of work area.

If a license holder works at a convention planning agency, he or she performs some duties similar to those of IMCs and is qualified to apply for the examination to obtain the IMC license. The duties of school nurses are not directly related with those of IMCs, but they are qualified to take the test to obtain the IMC license. Moreover, assistant nurses can take the examination to obtain the IMC license depending on their field of experience, as they are recognized to work in public healthcare or tourism practice although they do not have specialization on public healthcare or tourism (18).

For the development and growth of the international system of medical tour coordinators, it is recommended to introduce the employment quota system for license holders when hospitals in Korea enter into overseas markets.

The number of medical institutions in Korea operating business overseas increased by 14 cases from an accumulated 141 cases in 2015 to 155 cases in 2016 (20 new entry cases and 6 cases of business termination in 2016). In addition, the increase rate of the accumulated number of cases of entry into overseas market rose from 3% in 2014–2015 to 10% in 2015–2016 (19). Therefore, this study hereby predicts that if the employment quota system is introduced when hospitals in Korea operate business in another country, it can create more than 1,000 jobs. In order to revitalize the medical tourism industry, there is a need to upgrade the education of professional workers. For this to happen, the competitiveness of national qualifications should be strengthened through continuing on-site education after passing the IMC certificate exam.

## Conclusion

In this regard, this study hereby suggests the following considerations for the establishment of the policy to nurture human resources in the global medical tourism industry. First, a systematic support must be provided for training and education by establishing a systematic human resources nurturing platform that goes along with educational institutions, medical tourism companies through medical institutions to resolve the issue of supply-and-demand mismatch of medical tourism professionals, and establish the employment infrastructure connecting vocational training and duties to be performed in the field. Second, the employment quota system for the IMC license holders should be introduced with the National Assembly’s prior announcement of legislation that will be applied when hospitals are permitted to doing overseas business so that they can enter into the global market while creating jobs. Third, in order to lead the global healthcare market, it is necessary to strengthen the competitiveness of medical institutions and the supply and demand of professional manpower through the improvement of the national qualification examination system. This can be done by restructuring the qualification of the national IMC licensing examination.

## Ethical considerations

Ethical issues (including plagiarism, informed consent, misconduct, data fabrication and/or falsification, double publication and/or submission, and redundancy) have been completely observed by authors.
